# Life cycle environmental impact assessment of natural gas distributed energy system

**DOI:** 10.1038/s41598-024-53495-1

**Published:** 2024-02-08

**Authors:** Yakun Wang, Ting Ni, Bing He, Jiuping Xu

**Affiliations:** 1 College of Environment and Civil Engineering, Chengdu University of Technology, Chengdu, China; 2 Business School, Sichuan University, Chengdu, 610059 People’s Republic of China; 3 China Construction Eighth Bureau Second Construction Corporation Limited, 191 Nanxinzhuang West Road, Shizhong District, Jinan, Shandong 250024 People’s Republic of China

**Keywords:** Environmental sciences, Environmental social sciences

## Abstract

Natural gas distributed energy is recognized as a pivotal means to enhance energy efficiency and mitigate carbon dioxide emissions through localized energy cascading. Positioned as a key option for advancing the Sustainable Development Goals, this system optimizes energy utilization near end-users. While maximizing energy efficiency, it is imperative to address potential environmental challenges. A thorough, comprehensive environmental assessment, facilitated by the life cycle assessment method, proves instrumental in meeting this standard. Employing this method enables an intuitive grasp of the environmental strengths and weaknesses inherent in natural gas distributed energy within the power structure. This insight serves as a foundation for informed project decision-making, fostering the growth of the industry. We selected six environmental impact assessment categories based on the CML 2001 method, and conducted the life cycle analysis across four stages. China's inaugural natural gas distributed energy demonstration project was chosen as a model case, and an environmental impact assessment inventory was established, utilizing survey data and literature for comprehensive data collection and analysis. Results from case testing yield environmental impact assessment outcomes, with a specific sensitivity analysis for stages with notable environmental impact factors. The study underscores that the operation phase has the highest environmental impact, comprising 78.37% of the total combined environmental impact, followed by the fuel production phase. Comparative analyses with coal-fired and conventional natural gas power generation, based on dimensionless literature data, reveal that abiotic resources depletion potential is the primary contributor to the environmental impact of 1 kWh of electricity product, constituting 52.76% of the total impact value, followed by global warming potential. Concrete strategies have been outlined for decision-making in both the operational and planning phases of natural gas distributed energy projects. The strengthening of policies is pinpointed towards grid connection and scale expansion.

## Introduction

Natural gas stands as a prominent contemporary clean energy source, demonstrating cost-effectiveness and a state of relative maturity. Its utilization holds the potential to significantly diminish the environmental repercussions stemming from coal mining and production, contributing to the mitigation of climate change and fostering sustainable development. In recent years, the extensive utilization of fossil energy by humanity has led to significant environmental issues. The awareness of climate change and associated environment problems has been gradually increasing within the public and governments globally, resulting in the commitment of most countries to cut their emissions to a certain level^1^. Against this backdrop, there is a widespread acknowledgment that addressing environmental challenges necessitates a substantial augmentation in renewable energy generation^2^. Renewable energy has emerged as a key engine for expanding electricity production in China, with clean energy substitution playing an increasing role^3^. Although sustainable power production technologies such as solar and wind are rapidly developing, their implementation is challenging due to their intermittent nature^4^. Natural gas stands out as a clean and low-carbon fossil energy source, with its carbon content per unit of calorific value amounting to only 58% of coal and 74% of oil^5^. Moreover, the carbon emission reduction benefits of natural gas power generation are strikingly evident^6^. The global reserves of natural gas are exceedingly abundant, with shale gas emerging as a recently developed form of natural gas^7^, contributing significantly to the augmentation of natural gas supply. Simultaneously, its role is pivotal in enhancing the energy landscape and addressing environmental concerns^8^. Notably, in recent years, both the United States and the United Kingdom have transitioned from coal to natural gas and renewable energy sources, impacting carbon dioxide emissions reduction and substantially diminishing other air pollutants^9^. China stands as the world's foremost energy consumer and is anticipated to emerge as a significant demander of natural gas. Given the pivotal role of natural gas in China's decarbonization policy, it is projected that a substantial share of its future gas demand will be met through imports^10^.

Natural gas distributed energy systems have attracted significant attention for their low-carbon, flexible, and safe use of energy cascading close to the customer. Distributed natural gas energy is acknowledged for its superior energy efficiency and enhanced environmental performance compared to conventional coal-fired power generation, attributed to operational fuel distinctions^11,12^. Traditional natural gas-fired power generation and natural gas-fired distributed energy have relatively large differences. Although both utilize natural gas as the exclusive fuel, distributed power generation with natural gas attains gradient energy utilization, thereby enhancing energy efficiency and manifesting positive environmental impacts^13^. And natural gas distributed energy uses multiple small combustion engines for energy supply, which is more flexible and safer. Many distributed energy stations in foreign countries now rely on natural gas as the primary driving energy source. The energy utilization efficiency is higher than that of combined-cycle power plants, which can reach about 80% or more^14^. Natural gas has huge development potential, so vigorously developing distributed natural gas projects is still the mainstream of China's future energy structure adjustment. China’s shale gas industry has made great progress in recent years, and shale gas has the potential for sustainable development in terms of technology, economy and environment^15^. In comparison to alternative forms of distributed energy systems, those utilizing natural gas as a fuel exhibit several advantages. Firstly, their environmental performance surpasses others, as natural gas combustion does not produce dust^16^, thereby minimizing environmental impact. Secondly, their application is more versatile, contrasting with solar and wind energy, which are subject to geographical and climatic constraints^17^. Finally, shale gas, an unconventional natural gas resource, is developing rapidly in several countries around the world. It has also become an important strategic energy option for China. The development of the shale gas industry is conducive to China's ability to cope with its growing energy demand and reduce its dependence on imported fossil fuels^18^.

The Combined Cooling, Heating, and Power (CCHP) system, commonly referred to as tri-generation, is experiencing rapid global development due to its notable advantages, including high energy efficiency, low emissions, and enhanced reliability^19^. In comparison to conventional natural gas applications, the CCHP system has the capacity to significantly reduce greenhouse gas emissions, particularly carbon dioxide, thereby mitigating the risks associated with climate change and its environmental impact^20^. CCHP system overcomes the disadvantage of supplying a single form of energy and meets the energy needs of users. Electricity on the CCHP system is generated on-site, closer to the user's needs, thereby minimizing losses incurred during the transmission and distribution process^21^. Fundamentally based on the concept of energy cascading, the CCHP system addresses heat loads through the effective use of recovered heat. This design enables the system to achieve energy efficiencies exceeding 70%^22^, and in certain configurations, reaching as high as 90%, surpassing conventional stand-alone energy supply systems^23^. Remarkably, the CCHP system stands out as a smaller, more flexible, and decentralized energy supply system, providing enhanced reliability and stability throughout the entire process^24^.

In the initial phases of development, distributed energy systems primarily relied on natural gas-based combined heat and power system^25^. Subsequently, there was a widespread development of natural gas-fueled combined cooling, heating, and power (CCHP) systems, and the integration of distributed energy systems with renewable energy sources gradually emerged^26^. CCHP is the main form of utilization of natural gas distributed energy systems^27^. According to the definition of natural gas distributed energy by National Energy Administration in China, the distributed energy system (DES) mentioned in this paper refers to an energy system that utilizes natural gas as a fuel and employs cogeneration of cooling, heating and electricity. Technological advances are transforming the quality of life for billions of people, yet as the world's population grows and wealth, the environmental burdens of realizing these amenities are enormous. In this situation, it is particularly important to be able to assess and mitigate the environmental burdens involved in the systems.

The objective of this paper is to evaluate and analyze the comprehensive life cycle environmental impacts of the DES system in a real project using GaBi software. This aims to demonstrate the pivotal role of the DES system in attaining sustainable development goals. The rest of the research in this paper is as follows: Sect. 2 provides a review of the relevant literature. Section 3 introduces material and methods, including case background, research methodology and data sources. Section 4 provides a life cycle assessment of the case. Section 5 presents the results and performs a sensitivity analysis. Section 6 compares the evaluation results of the case of this paper with those of coal-fired and natural gas-fired power generation, and provides relevant recommendations for China's actual situation. Section 7 summaries some conclusions.

## Literature review

### DES from techno-economic perspective

Currently, in natural gas distributed energy systems, gas turbines and internal combustion engines are frequently employed in CCHP systems due to their high efficiency and compact size. Li^28^ compared the changing law of energy efficiency of natural gas distributed energy driven by gas internal combustion engine and gas turbine respectively, and based on it, gave the scope of application of the two in the actual popularization and application. Xiao et al.^29^ converted a gasoline engine into a gas engine with a CCHP system to form a small natural gas CCHP system. The study explored the waste heat and emission characteristics of the system, revealing that as the load increases, the amount of waste heat recovery grows, albeit with a decreasing proportion of total energy, maintaining an overall unit energy utilization above 80%. Advancements in technology have spurred interest in hybrid systems combining natural gas with renewable energy. Wang et al.^30^ proposed a configuration of a solar-assisted natural gas-fired CCHP system, and simulated the thermodynamic performance and the complementary characteristics of the coupling in different cases. It was found that the primary energy efficiency of the system could reach 71% under the designed operating conditions. Wang et al.^31^ presented the design and energy and economic multi-performance analysis of a solar-assisted CCHP distributed generation system. The article shows that under the design conditions, the energy efficiency of the cooling operation mode can reach 83.6% and that of the heating operation mode reaches 66.0%. Compared with conventional distributed energy sources without solar energy, the system consumes about 41% less natural gas per unit of energy. Yan et al.^32^ developed a thermodynamic model to simulate the performance of a natural gas CCHP system using an innovative approach combining a phosphoric acid fuel cell with solar technology. Results indicated that the integration of solar energy reduced natural gas consumption and enhanced overall efficiency by approximately 15%. In addition, Fang et al.^33^ devised a novel CCHP system integrating a three-stage organic Rankine cycle and a double organic flash cycle with liquefied natural gas as a heat sink. The article provided a thermodynamic analysis of the proposed CCHP system based on stipulated assumptions. In the multi-objective optimization outcomes, the system demonstrated an optimal efficiency of 80.49%, substantiating its superior performance.

Various scholars have conducted extensive studies on the economics of energy utilization in CCHP systems. Arsalis et al.^34^ have carried out thermodynamic analysis, fire-use analysis, and cost analysis of small-scale LNG-fueled CCHP plants to demonstrate the feasibility of this system as a substitute solution for distributed generation applications. Tookanlou et al.^35^ employed a particle swarm optimization algorithm to ascertain optimal hourly electricity and natural gas tariffs for CCHP systems, considering perspectives from both energy consumers and utilities. The study compared these tariffs with actual energy prices, confirming the interdependence of electricity and natural gas prices. Notably, operating the CCHP system in parallel with the distribution grid resulted in an 18% increase in the present value of revenues for distribution utilities. Yuan et al.^36^ developed an optimization model to enhance previous methods for optimizing the operation of a CCHP system integrating electricity and natural gas. The article compared the proposed method with others, validating its effectiveness, and designed four scenarios to assess the economics of electricity and natural gas. Hua et al.^37^ modeled a CCHP system for the coupled utilization of natural gas and geothermal energy, using a Beijing hotel as a case study. The proposed exergo-environmental cost method was applied to allocate the cost of multiple products, optimize the high-temperature flue gas allocation ratio, and evaluate energy consumption. The study concluded that the unit fire-environment cost is minimized when the flue gas allocation ratio is 0.63. Zhang et al.^38^ conducted a comparative economic analysis experiment using the National Natural Gas Distributed Energy Demonstration Project to assess the profitability of the CCHP project at different tariffs with fixed natural gas, heat, and cooling prices. They established a critical value model for calculating the operating profitability of CCHP projects, facilitating the calculation of the break-even tariff to optimize the operating strategy of CCHP units and maximize project revenue.

With the global emphasis on sustainable development, scholars are progressively incorporating environmental considerations into their research on energy systems. Chen et al.^39^ proposed a new multi-objective optimization model and tested it in the case of an integrated electrical and natural gas network for a CCHP plant. The case study demonstrates the model's effectiveness in enhancing the profitability and environmental performance of the system. Di Marcoberardino et al.^40^ investigated the environmental potential and economics of an innovative micro-DES based on a membrane reactor and a PEM fuel cell. The environmental analysis was accomplished using a life cycle assessment, while the economics were evaluated in terms of their maximum system cost that is cost-effective over their lifetime.

### CCHP from environmental perspective based on LCA

Life Cycle Assessment (LCA) endeavors to quantify the potential environmental impacts of a product, process, or service throughout its life cycle, encompassing direct and indirect emissions, as well as resource utilization from raw material acquisition through production, use, end-of-life treatment, recycling, and final disposal^41^. Over the past three decades, the methodology has evolved into a central tool for environmental management and decision support. Gradually, it has expanded to the level of sustainability analysis, introducing environmental, economic, and social evaluation indicators to enhance completeness and reliability. In the face of numerous sustainability challenges worldwide, a comprehensive assessment of relevant environmental issues can aid in addressing potential trade-offs for sustainability^42^. Our article focuses on studying the environmental impacts throughout the full life cycle process of the case, and the chosen method aligns with our current research problem. After years of development, more than 20 life cycle assessment (LCA) software have been developed worldwide, among which GaBi software is one of the most widely used LCA software at present. Developed by Thinkstep in Germany, its database integrates background databases from relevant research organizations and industries across various countries, comprising a total of more than 4000 available inventory data^43^.

As the application of Life Cycle Assessment is becoming more widespread in various industries, its Life Cycle Impact Assessment (LCIA) methodology is also evolving, as shown in Table 1. LCIA methods are classified into midpoint and endpoint methods based on differences in evaluation purposes. The endpoint method emphasizes ecological risks and human health end-effects more than the midpoint method. However, due to its complexity and high data requirements, the uncertainty of the results is slightly higher than that of the midpoint method, and its practical application is still challenging. In comparison to other midpoint methods, the CML2001 method reduces assumptions and model complexity, resulting in less uncertainty^44^. CML 2001 is a methodology published by the Center for Institute of Environmental Sciences at Leiden University in 2001. It has undergone development over the years, gaining widespread acceptance and making it suitable for comparing the results of this paper with other studies related to LCA.

**Table 1:** LCIA methods and characteristics.

Classification	Description	Characteristics
Midpoint method	CML 2001^52^	The method allows quantitative assessment of the eigenvalues, normalized values and weight values. The advantage of this method is that it reduces the number of assumptions and the complexity of the model
	TRACI^50^	The method contains 12 categories of impact factors, and there is a more complete database for each category, which is easier to use^45^. The output results are based on the product basis
Endpoint method	Eco-indicator 99^19,52^	The method is an endpoint method based on the Eco-indicator 95 method. The types of endpoint damage are mainly categorized as damage to human health, damage to ecosystems and resource depletion^46^
	ReCiPe^53^	The method combines midpoint and endpoint methods, allowing for easier interpretation and comparison between systems^53^. However, there may be differences between the results of the midpoint and endpoint methods^47^

There is a consensus that CCHP systems are more efficient than conventional energy generation and can reduce energy losses. Scholars are gradually focusing on whether CCHP has sustainable energy and how to improve the environmental friendliness of the system. Initially, researchers delved into the environmental aspects of Building Combined Heat and Power (BCHP) systems. Jing et al.^48^ developed a LCA model for a solar BCHP system. They applied this model to a BCHP system in an office building located in Beijing, China, and conducted a comparative analysis of the full life cycle energy and environmental performance under different operating strategies. Their findings indicate that, in terms of comprehensive performance, BCHP with the power load-following strategy yields superior benefits. Wang et al.^49^ proposed an optimization methodology for biomass gasification-based BCHP system combined with a life cycle inventory (LCI). Applying this method, a biomass BCHP case in Harbin, China was optimized to analyze the performance of multiple metrics such as cost, energy, and emission, and to evaluate its comprehensive performance. This study serves to illustrate that the integration of the optimization method with LCI is a robust and effective approach in the design of biomass BCHP systems.

Currently, some researchers have studied and compared the environmental performance of CCHP systems in different operating modes based on the LCA method. Yan et al.^50^ developed a parametric life cycle assessment framework using the TRACI methodology and simulated the energy generation and supply of a distributed CCHP system integrating office renewable energy and energy storage systems. The simulation results demonstrate that the system and the proposed technology have a lower environmental impact compared to conventional power generation. Regarding cost, the life cycle cost of the system exceeds that of conventional energy generation, with small and large offices proving to be more economical than medium-sized offices. Montazerinejad et al.^19^ introduced a novel solar CCHP system and applied LCA based on the Eco-indicator 99 methodology, complemented by exergo-environmental analysis to assess and comprehend the system's performance. Wang et al.^51^ proposed a Robust multi-objective optimization method integrated with LCA to minimize the environmental impact of a hybrid solar assisted natural gas CCHP system. Based on a case study to validate the optimization method, the environmental impact potential of the system was evaluated and the effectiveness of the optimization algorithm was demonstrated. Liu et al.^52^ conducted a life cycle assessment of DES to quantify their environmental impacts in comparison with conventional energy systems provided by natural gas and electricity. The GaBi software was employed for the LCA, Environmental impacts were evaluated using the CML methodology and the Eco-indicator 99 methodology, respectively, and sensitivity analyses were performed. The findings indicate that DES exhibit better life cycle performance during the use phase compared to conventional energy systems. The sensitivity analysis further showed that the environmental damage caused by DES can be reduced by optimizing natural gas and electricity consumption. Herrando et al.^53^ conducted a lifecycle assessment (LCA) of the solar combined cooling, heating and power (S-CCHP) system based on the ReCiPe method. The LCA results were then compared with conventional PV systems and grid-based systems. In addition, a sensitivity analysis was performed to analyze the impact of multiple metrics on the LCA results. The results of the study demonstrate that the S-CCHP system is more environmentally friendly and can reduce environmental impacts.

From the reviewed literature, it can be inferred that although there has been a gradual increase in the number of studies on DES, few studies have conducted detailed life cycle environmental assessment studies on DES systems. In addition, most of the hybrid systems in the cases in the literature are based on simulations and assumptions, while the case in this paper uses a real project of natural gas distributed energy CCHP. This paper offers a more realistic case reference, and provides valuable insights for product environmental impact studies.

### Life cycle modeling and testing of the DES

**Figure 1:**
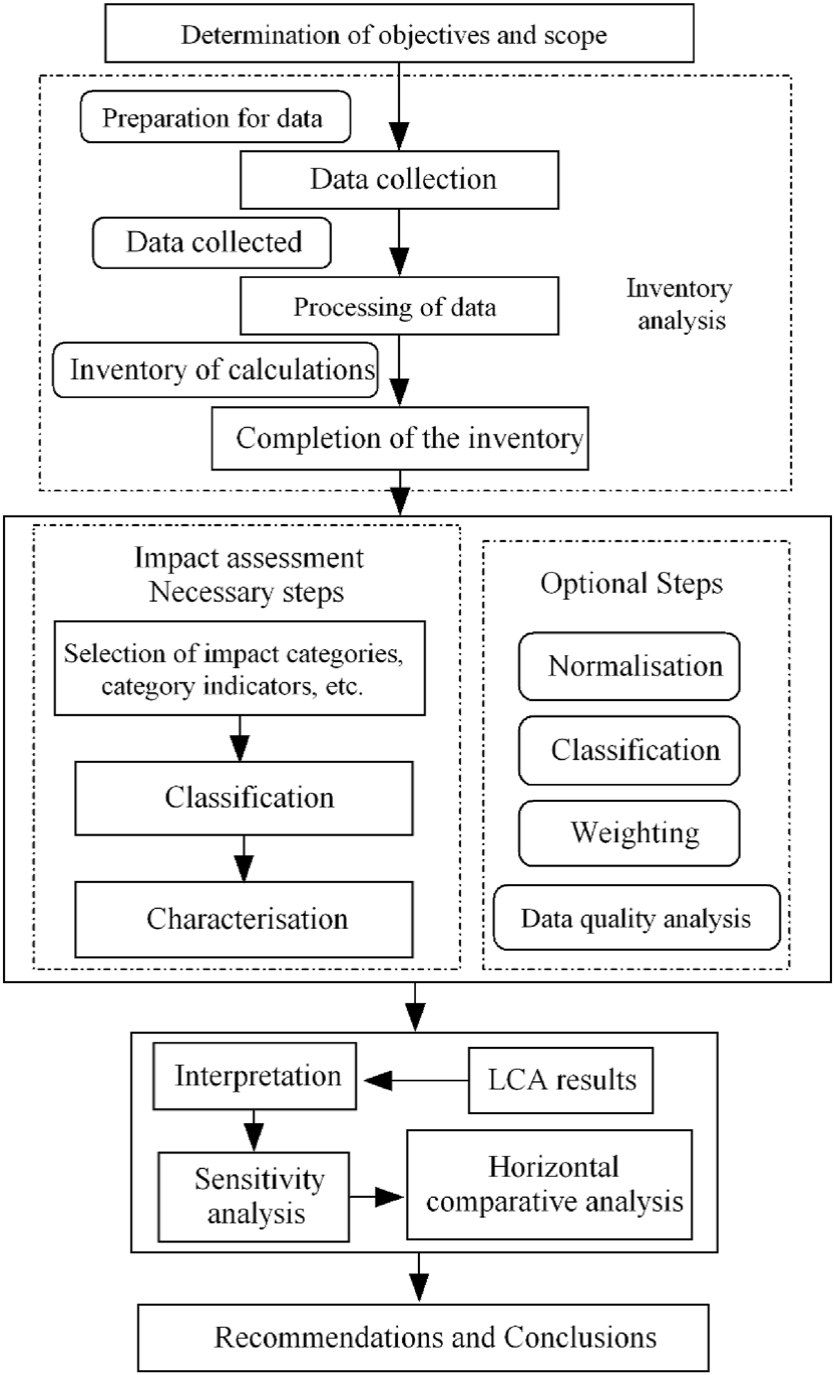
Flow chart of environmental impact assessment.

Given the increasing attention to reducing environmental pollution, it is crucial to assess the potential environmental impact of DES. It is necessary to carry out a full life cycle evaluation of DES to obtain their impact on the environment. Based on the guidelines of ISO14040^54^ and ISO14044^55^, LCA has the following four steps (Fig. 1). The determination of goals and scope mainly clarifies functional units and system boundaries, etc., which is the starting step of the entire life cycle assessment. Inventory analysis is the process of quantifying and inventorying all inputs and outputs involved in the entire life cycle of a product, process or activity. Life cycle impact assessment is the core link in LCA, which requires the inventory data to be calculated and quantified into different environmental impact types for evaluation. The interpretation is an analysis and summary of the three stages.

### System description and data preparation

The case study in this research pertains to the China Resources Snow Breweries natural gas distributed energy project in Sichuan province of China, which was recognized by the National Development and Reform Commission as the inaugural national natural gas distributed energy demonstration project. In the previous studies on this project conducted by other scholars^56^, life cycle analysis was adopted to compare the energy consumption and greenhouse gas (GHG) emissions of a natural gas-fired distributed generation project in China with five other scenarios. Their findings indicated that renewable natural gas possesses the potential to enhance energy efficiency and reduce GHG emissions. Their primary focus lay in assessing the project's overall performance of the project in terms of energy savings, GHG reductions, and economic efficiency. Different from existing research conducted in the project decision stage, this paper complements the environmental impact assessment of natural gas distributed energy based on the field research in the operation phase.

**Figure 2:**
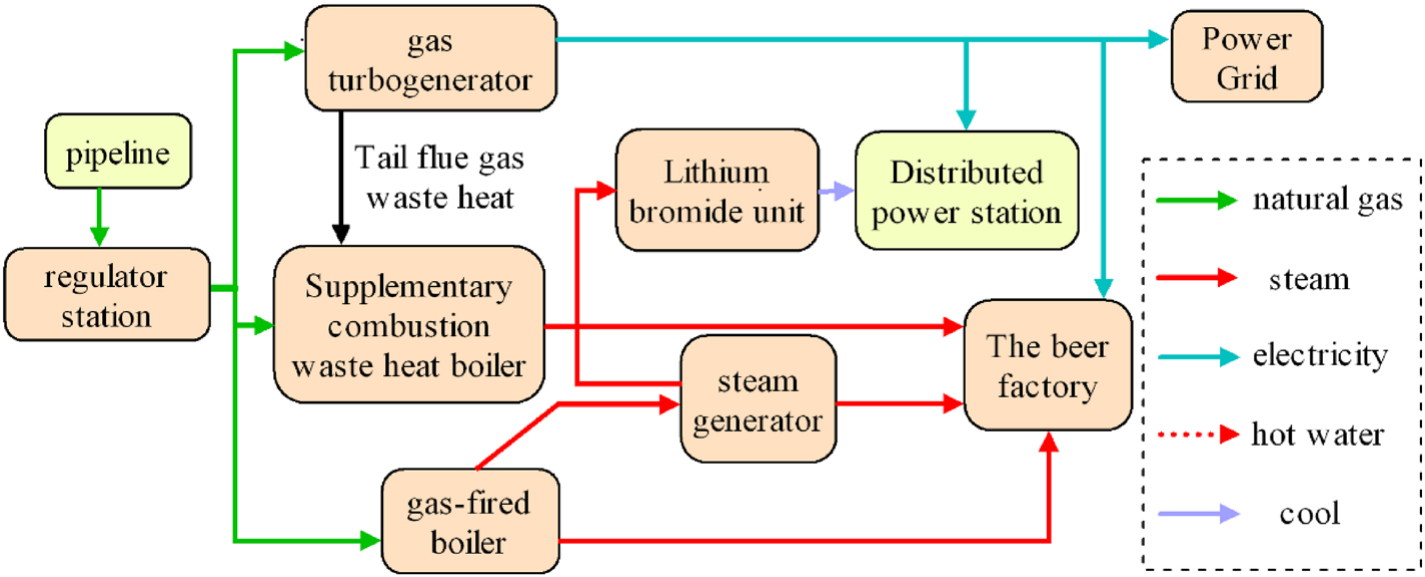
System configuration and operation schematic of the case.

The case examined in this paper is in an administrative district of Chengdu, China. It was developed to provide energy services to an industrial park in its vicinity. The case study employs a gas-steam cycle unit with 6 MW installed capacity. The project system configuration comprises a SolarT60 gas turbine, one supplementary-fired waste heat boiler, two 20 t/h gas boilers, a steam accumulator, a hot-water lithium bromide machine, and a 300 m^3^ heat storage water tank, and is not equipped with a steam turbine. The operation configuration principle of this case is shown in Fig. 2. According to National Energy Administration^57^, the annual steam supply of the case reaches 94,900 t (0.6–0.8 MPa/160–180 °C), and the annual cooling supply is 4900 GJ. The annual power generation is 31,154,200 kWh, the natural gas usage is 14,033,000 m^3^/a. Under annual average load conditions, natural gas consumption is 19,490,300 m^3^/h, and the low calorific value of the natural gas used is 33.93 MJ/m^3^, and density of 0.7145 kg/m^3^.

The data for the fuel production and fuel transport phases were mainly obtained from GaBi software. The fuel production phase calculates the energy consumption of the domestic natural gas production phase based on the 2021 China Energy Statistics Yearbook^58^. The fuel transport phase encompasses various aspects, including the volume of natural gas consumed, combustion emissions, gas leakage, electricity consumption, and pipeline distances traveled. Given the absence of specific data on consumables for the construction phase of this study, energy and material data for this phase were derived from comparable projects. During the operation phase of the project, we conducted field research at the project site in September 2020. This yielded measured data spanning the years 2016 to 2020 and annual data for each year of these years. The annual data for the whole operation period in this phase were computed as the average over the five-year duration of the study.

### Objective and scope definition

**Figure 3:**
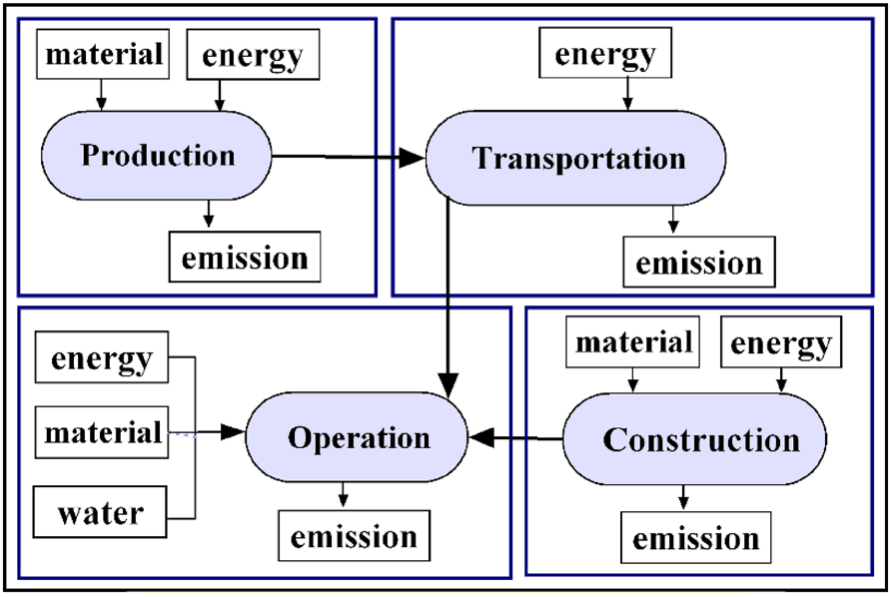
System boundary of the DES system.

The system boundary of the DES system in this study encompasses four phases: fuel production, fuel transport, project construction, and project operation as shown in Fig. 3. The decommissioning phase impact is not considered at this stage. At present, life cycle assessments of power systems typically employ unit power generation as the functional unit. Therefore, this paper selects the natural gas distributed energy output of 1 kWh of electricity as the functional unit.

### Environmental impact assessment

#### Selection of six impact categories

From Section 2.2 we know that CML2001 approach is widely used by scholars due to minimized assumptions and model intricacies with decreased uncertainty. There are 11 categories. However, according to the different evaluation systems and the differences in the local environment, the number of selected impact categories vary from four^59^ to ten^60^. Through our environmental investigation of the case project site, we have found that the region tends to experience persistent pollution processes every late winter with poor meteorological dispersion conditions and continuous cumulative impacts of pollutant concentrations. In addition, the DES system studied in this paper is fueled by natural gas, and the combustion process emits pollutants such as CO_2_ and NO_X_. Our analysis and assessment of the categories with high environmental impacts can provide an overall picture of the environmental impacts of the project. Therefore, this paper selected six impact categories of Global Warming Potential (GWP), Eutrophication Potential (EP), Acidification Potential (AP), Photochemical Ozone Creation Potential (POCP), Abiotic Resources Depletion Potential (ADP), and Human Toxicity (HTP) as indicators, considering their significance in the study.

#### Inventory analysis in four phases

##### Fuel production phase

The inventory analysis for this stage considers the primary and secondary energy consumption consumed to produce natural gas, as well as the emissions generated during the process. Emission coefficients of different energy sources with different utilization modes were referred to Xu^61^. The results of direct emissions from the fuel production phase are shown in the Table 2.

**Table 2:** Inventory of emissions from the production phase of fuels (kg/m^3^).

Types	CO_2_	CH_4_	SO_2_	CO	NO_2_	PM10	VOC	N_2_O
Emissions	3.41E−01	1.21E−04	9.21E−04	8.65E−04	5.57E−04	2.83E−04	1.14E−04	4.24E−06

##### Fuel transport phase

This phase of the inventory analysis collects elements such as the amount of natural gas consumed as well as its combustion emissions, leaked natural gas (predominantly methane), electricity consumed, and the distance traveled by pipeline. The findings of Ou et al.^62^ showed that pipeline transport of 1 kg of natural gas consumes 7.66E−04 MJ of natural gas and 6.67E−06 MJ of electricity per kilometer. The types of emission pollutants considered for the natural gas burned as fuel at this stage and the emission factors refer to the research data of Song^63^. Given the inherent likelihood of methane leakage during transport, the methane leakage is set at 0.3% of the total transported volume^64^. The results of direct emissions during the pipeline transportation phase are shown in the following Table 3.

**Table 3:** Inventory of energy consumption and emissions during the pipeline transportation phase.

Type of energy consumption	Energy consumption (MJ/m^3^)	Direct emissions	Emissions (kg/m^3^)
Natural gas	4.88E−01	CO_2_	2.85E−02
Electricity	4.25E−03	CH_4_	2.32E−05
/	/	N_2_O	9.50E−07
/	/	NO_x_	2.62E−05
/	/	CO	3.48E−05
/	/	PM10	4.91E−07
/	/	SO_x_	4.12E−06

##### Project construction phase

The inventory analysis of the construction phase of the DES focuses on the energy consumed, raw materials, production of raw materials, and transport of indirect energy consumption and emissions brought about by the system during the construction process. As specific consumable data for the construction phase were not available, this paper draws upon research data by Zheng^65^ to determine energy consumption and materials. The mode of transport of consumables in the construction phase consists of 80% road and 20% rail. The results of direct emissions during the construction phase are shown in the Table 4.

**Table 4:** Emission inventory in construction phase (kg/kWh).

Types	CO_2_	CH_4_	SO_2_	CO	NO_2_	PM10	VOC	N_2_O
Emissions	1.68E−03	5.37E−07	7.13E−06	1.02E−06	1.2E−06	3.25E−08	4.49E−07	9.13E−08

##### Project operation phase

In this phase, the primary consideration is energy consumption, mainly the amount of natural gas consumed for system operation, while the focus regarding emissions is atmospheric emissions resulting from natural gas combustion. It is important to note that, due to the products of the energy station not being solely electricity, but also including both cooling and heating products. Therefore, we convert the energy of heating and cooling into electricity, and further convert it into the quantity of natural gas consumed. Applying the first law of thermodynamics and converting the total energy from cooling and heating into a unified electricity unit, the total energy amounts to 10.451 million kWh. Throughout the year in case project, the consumption of natural gas totaled 14.033 million m^3^, and the production of one unit of electricity during the operating phase necessitates the consumption of 0.134 m^3^ of natural gas. Inventory emissions during the operational phase are shown in the Table 5.

**Table 5:** Emission inventory in operation phase (kg/kWh).

Types	CO_2_	CH_4_	SO_2_	CO	NO_2_	PM10	VOC	N_2_O
Emissions	1.81E−01	2.23E−05	2.01E−05	3.33E−04	2.51E−04	4.70E−07	2.69E−05	9.13E−06

#### Characterization results

Characterization consists of assigning the environmental disturbances emitted during the life cycle of the object of study to the corresponding impact categories and transforming them into indicators that can represent potential impacts on the environment. Common impact factors for several environmental impact categories and their characterization factor values are shown in Table 6.

**Table 6:** Common impact substances and their characterization factor values for environmental impact categories.

Impact category	Impact substance	Characterization factor value	Unit
GWP	CO_2_	1	kg CO_2_.eq
	NO_x_	5	
	CH_4_	28	
	CO	1	
EP	PO_4_^3−^	1	kg PO_4_^3−^.eq
	Ammonia	0.35	
	Nitrate	0.1	
	NOx	0.13	
	Phosphorus	3.06	
AP	SO_2_	1	kg SO_2_.eq
	NO_x_	0.5	
	NH_3_	1.6	
	HCL	0.749	
POCP	C_2_H_4_	1	kg C_2_H_4_.eq
	Methane	0.006	
	CO	0.027	
	SO_2_	0.048	
ADP	Run of mine coal	11.9	MJ
	Crude oil	42.8	
	Natural gas	46.2	
	Hard coal	26.3	
HTP	DCB	1	kg DCB.eq
	Carbon Tetrachloride	0.73	
	Chloromethane	0.02	

Our study calculated and obtained the results of characterization of the six impact types over the life cycle of the study case according to Eq. (1), and the results were displayed in Table 7.

1 $${EIP}_{j}=\sum {EF}_{j}(i)\times M(i)$$

where ${EIP}_{j}$ is the contribution of the product system to the ${j}$th environmental impact type, that is the value of the characterizing score, eq/kWh, ${EF}_{j}(i)$ is the characterizing factor for the ${j}$th environmental impact of the ${i}$th emitting material, eq/kg, and $M(i)$ is the number of emissions of the ${i}$th material, kg/kWh.

**Table 7:** Characterization results for the study cases (1 kWh).

Environmental impact categories	Fuel production	Fuel transport	Project construction	Project operation	Life cycle
GWP (kg CO_2_.eq)	3.71E−02	8.82E−03	2.09E−03	2.16E−01	2.64E−01
EP (kg PO_4_^3−^.eq)	5.63E−06	2.82E−06	3.45E−07	2.25E−05	3.13E−05
AP (kg SO_2_.eq)	3.56E−05	3.64E−06	8.62E−07	5.11E−05	9.12E−05
POCP (kg C_2_H_4_.eq)	1.04E−05	9.78E−07	1.63E−07	4.72E−06	1.63E−05
ADP (MJ)	4.61E−01	1.65E−01	2.69E−02	3.18E−00	3.84E−00
HTP (kg DCB.eq)	5.18E−04	6.75E−05	4.05E−04	1.22E−03	2.25E−03

#### Normalization results

After characterization, a standardized methodology processes the results of the various environmental impacts to establish a benchmark for comparisons and to determine the contribution of each impact type. In this paper, we use the global environmental impact benchmark values from the CML2001 impact assessment methodology as the normalization factor, with the year 2000 chosen as the base year, in standard human equivalents. Equation (2) for standardization is given below and the standardization results for the study cases were shown in Table 8.

2 $${NEIP}_{j}={EIP}_{j}/{NF}_{j}$$

where ${NEIP}_{j}$ is the standardization value of the ${j}$th environmental impact in the product system; ${EIP}_{j}$ is the results of characterization of environmental impact type j; ${NF}_{j}$ is the normalization factor.

**Table 8:** Environmental impact values after normalization of study cases (1 kWh).

Impact category	Fuel production	Fuel transport	Project construction	Project operation	Life cycle	Percentage (%)
GWP	8.79E−16	2.09E−16	4.95E−17	5.11E−15	6.25E−15	32.65%
EP	3.56E−17	1.78E−17	2.18E−18	1.42E−16	1.98E−16	1.03%
AP	1.48E−16	1.52E−17	3.61E−18	2.13E−16	3.81E−16	1.99%
POCP	2.82E−16	2.65E−17	4.42E−18	1.28E−16	4.43E−16	2.31%
ADP	1.21E−15	4.34E−16	7.07E−17	8.36E−15	1.01E−14	52.76%
HTP	4.07E−16	5.31E−17	3.18E−16	9.61E−16	1.77E−15	9.25%

#### Weighting results

By assigning different weights to each environmental impact category, the quantitative results of the integrated environmental impacts over the life cycle of the case can then be obtained through weighted calculations. These values reflect the degree of impact of a certain environmental impact on the entire ecological environment, calculated by Eq. (3). The results were shown in Table 9.

3 $${TEIP}_{j}={NEIP}_{j}\times {WF}_{j}$$

where ${TEIP}_{j}$ is the weighted result value for category j environmental impacts; ${NEIP}_{j}$ is the normalized result value for category ${\text{j}}$ environmental impact types; and ${WF}_{j}$ is the weighted value for category j environmental impact types.

**Table 9:** Weighted environmental impact values for study cases (1 kWh).

Impact type	Fuel production	Fuel transport	Project construction	Project operation	Life cycle
GWP	8.17E−15	1.94E−15	4.60E−16	4.75E−14	5.81E−14
EP	2.35E−16	1.17E−16	1.44E−17	9.37E−16	1.31E−15
AP	9.03E−16	9.27E−17	2.20E−17	1.29E−15	2.32E−15
POCP	1.83E−15	1.72E−16	2.87E−17	8.32E−16	2.88E−15
ADP	8.47E−15	3.03E−15	4.95E−16	5.86E−14	7.07E−14
HTP	2.89E−15	3.77E−16	2.26E−15	6.82E−15	1.25E−14
Sum of categories	2.25E−14	5.73E−15	3.28E−15	1.16E−13	1.48E−13
Percentage (%)	15.55%	3.87%	2.21%	78.37%	100%

## Discussion

The environmental impacts have been estimated following the CML 2001 impact assessment method. We analyze the results of this paper in this section, based on which a sensitivity analysis is performed. Then we compare the assessment results of this paper with those of coal-fired and natural gas-fired power generation to accurately assess the environmental advantages of natural gas distributed energy. Meanwhile, countermeasure suggestions suitable for China's current situation are put forward for the obstacles encountered in China's development, and we hope to promote the realization of the industry's sustainable development.

### Modeling result analysis

#### Environmental impacts of the DES

The largest contribution to the GWP, AP and EP impact potential of the case is from the operational phase of the project, followed by the fuel production phase. This is mainly due to the large amount of natural gas consumed and the large amount of CO_2_ and NO_X_ emitted during the operation phase of the project. The case's contribution to the ADP impact potential comes mainly from the use of natural gas, coal, and oil. The operational phase of the project contributes the most to the ADP impact potential, most notably because it consumes a large amount of natural gas and has the largest natural gas characterization factor value in the ADP. This is followed by the fuel production phase. The primary contribution to the POCP impact potential from the case arises from emissions of substances like N_2_O and SO_2_. The most substantial contribution is attributed to the fuel production phase, followed by the operational phase of the project. The largest contribution to the HTP impact potential is during the operational phase of the project, where emissions of polycyclic aromatic hydrocarbons from natural gas combustion are a significant contributor to the HTP impact potential. The fuel transportation phase and the project construction phase contribute less to the impact potential of each impact category.

After characterization, the units of different impact types are not the same, and normalization and weighting can provide an inter-comparable benchmark for judging the level of environmental impact hazard over the life cycle. In addition, subsequent comparisons of cases from different literatures can be made based on this. By analyzing the standardized results, we have been concluded that ADP has the largest environmental impact during the whole life cycle of the product of the study case outputting 1 kWh of electricity, followed by GWP, and EP has the least impact. Reducing natural gas leakage and improving the efficiency of energy use in actual production and operation can reduce the environmental impact values of ADP and GWP.

Tables 9 and 10 reveals that the phase with the greatest environmental impact is the operational phase of the project, which accounted for 78.37% of the total value of the combined environmental impact, followed by the fuel production phase, which accounted for 15.55%. The type with the highest environmental impact throughout the life cycle process is ADP, followed by GWP. The primary contributors to the most severe environmental impacts during the operational phase of the project are the substantial emissions from combustion and the consumption of natural gas. Focusing on the integrated environmental impacts over the life cycle of DES starts with the operational phase of the project.

**Table 10:** Values of environmental impacts at different stages and their proportions.

Sensitivity factors	Sensitive factor value	Rate of change	Sensitivity factor value after change	Indicator after change	Rate of change of integrated environmental impact	Sensitive factor
C1 power generation efficiency (%)	31.5%	− 10%	28.35%	1.61E−13	8.783%	0.75
		+ 10%	34.65%	1.36E−13	− 8.108%	
C2 natural gas consumption (m^3^/kWh)	0.137	− 10%	0.123	1.37E−13	− 7.432%	0.62
		+ 10%	0.151	1.59E−13	7.297%	
C3 energy consumption (MJ/kWh)	0.43	− 10%	0.387	1.42E−13	− 4.054%	0.34
		+ 10%	0.473	1.53E−13	4.237%	

#### Sensitivity of impact factors

Sensitivity analysis in life cycle evaluation aims to assess the impact of variations in data and parameters on the results and conclusions. The results of sensitivity analyses are indicative of the life cycle assessment 's reliability and precision^66^. In this paper, sensitivity analysis is used to study the extent to which parameter changes affect the results to obtain a basis for improving environmental impacts. The selection of parameters considers those parameters that have a greater impact in the staged results obtained in the case for the category of environmental impact under study.

In the previous section, the two phases of the case with the greatest environmental impacts were identified through the results of the environmental impact assessment as the operational phase of the project and the fuel production phase. Through the analysis, it is found that when the values of the three factors of system power generation efficiency in the operation stage, natural gas consumption, and energy consumption level in the fuel production stage change, the results of the list of cases will follow the changes, which will cause changes in the magnitude of the impact on the environment. Therefore, this paper will select these three factors for sensitivity analysis, numbered C1, C2, C3, each subjected to a ± 10% value change to assess their combined environmental impact (Table 10).

In summary, the high sensitivity of power generation efficiency and natural gas consumption is stems from the operation phase 's substantial contribution of 78.37% to the total environmental impacts across the study case's entire life cycle. Enhancing power generation efficiency leads to greater energy utilization efficiency and increased electricity output, while reducing natural gas consumption decreases direct emissions during the operation phase in the same situation. Therefore, future research and development efforts should prioritize improving power generation efficiency and enhancing overall energy utilization efficiency to effectively reduce the environmental impacts of natural gas distributed energy throughout its life cycle. Although the sensitivity factor of energy consumption in the fuel production stage is relatively smaller than the first two, reducing the production energy consumption and improving the extraction level of the upstream oil and gas industry is also one of the effective measures to reduce the life cycle environmental impacts of the study case.

### Comparative analysis

#### Coal-fired power generation

In 2022, coal power constituted 43.8% of the installed capacity, yet it contributed 58.4% to the country's total power generation^67^. Coal-fired power generation (CPG) systems pose significant environmental challenges. To highlight the environmental advantages of DES compared to coal, this paper conducts a comprehensive analysis of literature on the life cycle assessment of CPG. Given the extensive literature available, a focused approach was adopted, screening for studies using the CML2001 impact evaluation method with a functional unit of 1 kWh of electricity. The literature considered spans from 2011 up to date to facilitate a direct horizontal comparison of results and is presented in Table 11.

**Table 11:** Main references and their characterization results for CPG.

Literature	GWP kg CO_2_ eq/kWh	AP g SO_2_ eq/kWh	EP g PO_4_^3−^ eq/kWh	POCP g C_2_H_4_ eq/kWh	ADP MJ/kWh	HTP g DCB eq/kWh
Petrescu et al.^68^	0.97	0.49	1.28	0.2	9.82*	3.41
	0.50	1.61	1.75	0.25	14.13	19.55
	0.49	4.57	1.73	0.27	15.23	15.27
	0.40*	0.49	1.21	0.26	13.75	19.84
Zhou^69^	1.62	11.5***	/	/	/	13.04
	1.59	9.61	/	/	/	11.02
	0.65	7.57	/	/	/	8.94
	0.96	7.89	/	/		9.32
Nugroho et al.^70^	1.06	5.89	2.62	/	15.8***	/
Wang^71^	2.81	0.677	0.98	/	10.48	10.20
	2.97	0.714	1.04	/	11.04	11.02
Li et al.^72^	0.92	4.62	0.53*	0.34	/	21.03***
Yue et al.^73^	3.26***	/	/	2.74***	/	10.02
Zhang^74^	1.12	5.1	/	/	/	/
Atilgan et al.^75^	1.062	10.8	11.9***	0.48	13.5	/
	1.126	6	2.3	0.33	15.1	/
Liang et al.^76^	0.813	0.379*		0.033*	/	2.87*
	0.971	0.097		0.0854	/	3.72
	0.83	1.32	/	0.0783	/	3.16
	0.801	1.34	/	0.0506	/	3.24
Morrison^77^	1.16	/	/	2.33	/	/
Siddiqui et al.^78^	0.8211	2.5	/	0.129	/	/
Mean value	1.22	4.16	2.53	0.54	13.2	10.35
The case study	0.264	0.09	0.03	0.16	3.84	2.25

***Represents the maximum values of the characterization results of each impact category from the literature. *Represents the minimum values of the characterization results of each impact category from the literature.

The study cases in this paper compared with CPG results, and it was evident that the minimum values for environmental potentials in the other five impact types exceeded those in this paper, except for POCP. For the GWP, EP, AP, POCP, ADP, and HTP impact types, the mean impact potentials of CPG are 4.6, 84.3, 46.2, 3.4, 3.4, and 4.6 times higher than those in this paper, respectively. It can be seen from the table that the contribution of CPG to EP and AP is very prominent, and the most direct reason for this result is mainly due to the difference in fuel, which produces large amounts of carbon oxides, nitrogen oxides, sulfur compounds, and suspended particulate matter when coal is burned. Natural gas is a clean energy source, the gases emitted after combustion are mainly methane, ethane, propane, isopentane and carbon dioxide, with very little soot emission. Although natural gas also contains a small amount of sulfur, only a trace amount of sulfur dioxide will be produced after combustion, far less than the emissions of coal power. The projects in the case study also use low nitrogen combustion technology, which can further reduce nitrogen oxide emissions. In addition, the different efficiency of energy utilization can also have an impact, DES follows the stepwise utilization of energy, and the comprehensive energy utilization can reach 70% to 90%^79^.

#### Conventional natural gas power generation

The key differences of conventional and distributed natural gas-fired power generation lie in scale, proximity to end-users, and the integration of combined heat and power technologies in distributed natural gas systems. A comparative assessment of the environmental performance of gas-electricity and the results of this paper can help determine the superior form of utilization. Like the inclusion and exclusion criteria used before, we filter literature that uses the CML2001 impact evaluation method with a functional unit of 1 kWh of electricity. We consider literature published from 2011 to present, and display the evaluation results from various references meeting these criteria in Table 12.

**Table 12:** Main references and their characterization results for gas and electricity.

Literature sources	GWP kg CO_2_ eq/kWh	AP g SO_2_ eq/kWh	EP g PO4_4_^3−^ eq/kWh	POCP g C_2_H_4_ eq/kWh	ADP MJ/kWh	HTP g DCB eq/kWh
Atilgan^75^	0.388	0.15*	0.04*	0.03*	6.3*	16.5
Karapekmez^80^	0.56***	0.65	/	/	/	/
Ozturk et al.^81^	0.54	0.62	0.0767	/	/	41***
Singh et al.^82^	0.459	0.453	/	0.079	/	/
Bicer et al.^83^	0.410	0.34	0.071	0.62***	/	15.59
Agrawal et al.^84^	0.455	/	/	/	/	/
Martin et al.^85^	0.503	0.92***	0.14	/	9.38	/
	0.524	0.83	0.15***	/	9.87***	/
Fadeyi et al.^86^	0.365*	0.42	/	/	/	/
Binita et al.^87^	0.502	0.56	0.08	/	/	8.4*
Mean value	0.43	0.54	0.08	0.233	6.42	16.25
The case study	0.264	0.09	0.03	0.16	3.84	2.25

***The maximum values of the characterization results of each impact category from the literature. *The minimum values of the characterization results of each impact category from the literature.

One of the reasons for the large differences in the characterization results between the different literatures for the same impact type is that there are differences in the efficiency and unit conditions of the different gas and electricity projects. The second is that natural gas comes from different sources in different countries, which is related to the resource endowment of the location of the gas and electricity projects. If the demand for natural gas imports is high, it is usually transported by marine transportation vessels, and there are differences in the results of the life cycle assessment between transportation vessel transportation and pipeline transportation.

The difference between the resultant values of this paper's case and those of conventional natural gas power generation (CNGPG) is less pronounced than with CPG. CNGPG exhibits larger overall characterization results, particularly in terms of GWP, EP, AP, POCP, ADP, and HTP impact types. The reason for this difference is that the research case in this paper is compared with traditional natural gas power generation. Although both use natural gas as their sole fuel source, CNGPG lacks secondary energy utilization. The production of natural gas distributed energy results in a smaller lifecycle output of materials energy consumption per unit of electricity. Moreover, the distribution of energy in natural gas distributed energy is closer to end-users, reducing energy losses in distribution and obviating the need for large-scale transmission facilities, which helps reduce the investment and the life cycle of energy consumption and emissions.

#### Comprehensive comparison

**Figure 4:**
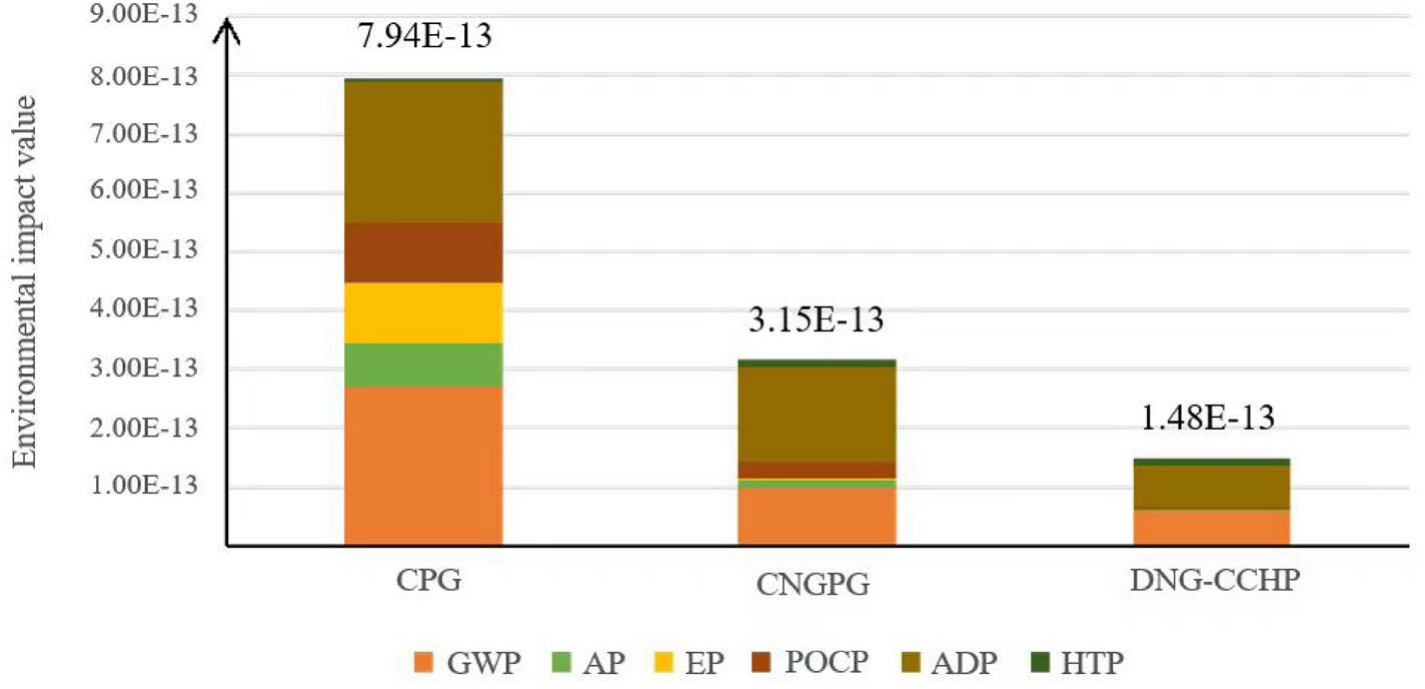
Combined environmental impact value of the three power generation approaches.

Figure 4. illustrates the combined environmental impacts of CPG and CNGPG for 1 kWh of electricity output, achieved through a normalization and weighting process. In CPG systems, the largest environmental impact is attributed to GWP, followed by ADP. In contrast, for CNGPG systems and the DES system discussed in this paper, the primary environmental impact is ADP, followed by GWP. The environmental impact of the thermal power industry is mainly manifested with energy consumption and the emissions of various greenhouse gases. The combined environmental impact value of the case in this paper is 18.63% of coal power generation; 46.98% of natural gas power generation. This suggests that CPG has the most significant environmental impact, followed by CNGPG. The DES system in this paper exhibits notable advantages. Therefore, under the national carbon peaking and carbon neutrality goals, natural gas distributed energy has a very good development prospect and environmental advantages when transforming the energy structure from the results of the environmental impact assessment.

Although DES has the advantages of low carbon, stability, and flexibility, its development is restricted by factors such as resource endowment and economic cost and cannot completely replace the existing power generation system. Global natural gas reserves are declining, and in the absence of breakthroughs in shale gas, natural gas distributed power generation needs to be used as one of various forms of power generation to protect people's livelihood. With the advancement of science and technology, the application of biomass, geothermal, solar and other renewable energy sources integrated with natural gas to generate electricity will be beneficial to the sustainable development of the natural gas industry.

### Recommendation

#### Strategies for the DES projects

The sensitivity analyses conducted in this case identified power generation efficiency and natural gas consumption during the operation phase as the most sensitive factors. And the LCA-weighted results also indicated that the operation phase had the highest environmental impact value in the whole life cycle process of the study case. Therefore, we offer the following recommendations for projects that are in operation.

*Use clean energy and advanced technology*. We recommend the use of clean energy sources and raw materials, the adoption of advanced technology and equipment, and the enhancement of management practices^88^. This approach aims to achieve comprehensive resource utilization, pollution reduction at the source, improved resource efficiency, and a reduction of hazards to both human health and the environment.

*Collaborate with gas enterprises*. Jointly operate the project with gas enterprises to ensure the effective supply of gas^89^. The normal operation of gas units requires the effective cooperation of gas enterprises, which will jointly construct and operate the project, improve the gas infrastructure, and ensure that the units have enough gas sources.

*Implement energy monitoring and control*. Developing energy demand and consumption monitoring is crucial. Real-time monitoring and data analysis can help optimize unit operations^90^. We recommend the creation of an intelligent control application platform for energy stations to enable intelligent production regulation based on demand and production supply dynamics.

*Enhance operation and maintenance*. To maintain reliable operation, it is vital to strengthen the operation and maintenance of core equipment, such as gas turbines. At the same time, the economic, social, and safety aspects associated with gas facilities should be strictly regulated^91^. Regulate the use and management of infrastructure and improve the system.

For projects in the planning stage, we put forward three points for the reference of the relevant enterprises:

*Local government cooperation*. Actively collaborate with local governments to create an integrated energy system that aligns with local conditions. Leveraging local natural resources, such as solar energy, geothermal energy, wind energy, and hydropower, can lead to a comprehensive and intelligent energy system^92^. This can enhance energy efficiency and the security of supply.

*Optimize system configuration*. Conduct adequate research and validation from the project planning stage to determine the most suitable system configuration and operation mode^93^. Maximize comprehensive energy utilization efficiency and power generation efficiency.

*Research and development*. Relevant enterprises should prioritize research and development in core technologies and instruments. This includes manufacturing and technological upgrades of essential instruments like gas internal combustion engines and gas turbines. Additionally, strengthening research in areas such as system integration and optimized operation is essential.

#### Policy implications

**Figure 5:**
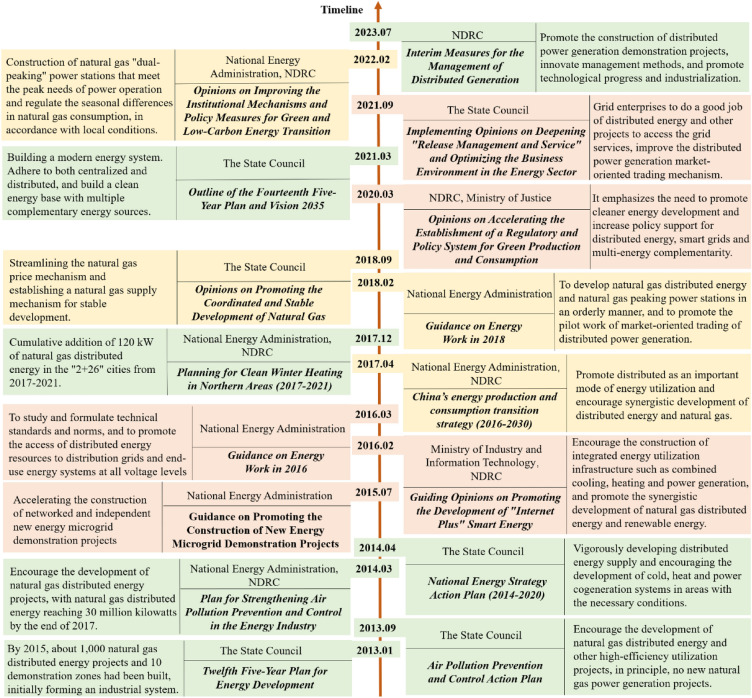
Specific contents on distributed natural gas in the related policy documents from 2013 to the present in China. Contents in yellow boxes are related to the consumption structure of the energy market and the energy price. Contents in red boxes are related to the construction acceleration of grid connection. Contents in green boxes are related to the state's efforts to strengthen the industrial development of natural gas distributed energy projects. NDRC is short for National Development and Reform Commission in China.

From the experience of foreign countries' development, government-issued policy support plays a crucial role in facilitating the rapid growth of natural gas distributed energy. In 2011, four ministries and commissions jointly issued the "Guidance on the Development of Natural Gas Distributed Energy" guidance document, one after another in the energy strategy plan are proposed to vigorously develop natural gas distributed energy, "Twelfth Five-Year" plan, "Thirteenth Five-Year" plan, "Fourteenth Five-Year" plan of natural gas distributed energy as a need to promote the focus of the field. Since 2013, the National Energy Administration, the National Development and Reform Commission, the State Council and other departments have issued a series of supportive policy documents to promote the development of natural gas distributed energy, and some of the key related policies are shown in the Fig. 5.

A detailed interpretation of the figure reveals that China's policy direction for promoting natural gas distributed energy development primarily focuses on several aspects. These include increasing the proportion of natural gas consumption by adjusting the consumption structure of the energy market. Reduce the operating costs of natural gas distributed energy, thereby enhancing the competitiveness of the industry. Focusing on the preparation of natural gas distributed energy to be connected to the grid. Analyzing the policy direction alone showcases the country's determination and commitment to developing natural gas distributed energy. However, the existing policy system has deficiencies, hindering the standardized development of natural gas distributed energy. We have provided relevant recommendations for the future policy direction, aiming to contribute to the improvement of the situation.

*Enhance policy implementation rules*. Strengthening the implementation rules on policy implementation in conjunction with fiscal, taxation, finance and pricing. Taking the Implementing Rules for Natural Gas Distributed Energy Demonstration Projects as an example, the document proposes to have certain investment incentives or subsidized interest rates for natural gas distributed energy projects, but it is not clear how the investment incentives and subsidized interest rates are to be implemented.

*Harmonized of electricity subsidies*. China's existing electricity pricing policy for natural gas distributed energy generation lacks a unified subsidy standard for Internet access. The government should proactively implement measures, including financial subsidies and gas price concessions, to resolve price conflicts in natural gas power generation^94^.

*Inclusion of technical standard specifications*. Enhance the policy content by incorporating more relevant technical standards and norms. The grid connection of natural gas distributed energy power generation is a problem^95^. The Electricity Law emphasizes that enterprises with power generation qualifications must not only meet the criteria for connecting to the electricity grid but also obtain the consent of power grid enterprises. The miniaturization, multi-purpose, and fragmented characteristics of natural gas distributed energy pose challenges in meeting legal requirements for grid connection.

*Enhance local policies and implementation*. Expand the scope of policy implementation by introducing more targeted local policies. The development of natural gas distributed energy varies across different provinces and municipalities, and policies are often generalized. Currently, aside from the more developed North, Shanghai, Guangzhou, and the Yangtze River Delta region, many provinces lack clear preferential policies^96^. To genuinely promote institutional reform, it is crucial to effectively implement supporting policies in provinces, municipalities, and regions.

### Limitation

Due to the study's inherent scope limitations and the unavailability of precise data in certain sections, various areas still pose challenges, prompting the need for further investigation. Despite meticulous efforts to collect the latest statistical data, some information had to be substituted with data from earlier years, particularly in a less-explored subset. Consequently, additional field research is imperative to enhance the data quality in these specific domains.

Moreover, it is crucial to note that this study revolves around an operational project, and the absence of data from the decommissioning phase hinders the strict comprehensiveness of our life cycle assessment. To address this gap, future endeavors should focus on obtaining relevant data during the decommissioning phase.

## Conclusion

This paper chose China's inaugural natural gas distributed energy demonstration project as a model case, and established an environmental impact assessment inventory across four stages. Results from case testing yield environmental impact assessment outcomes, with a specific sensitivity analysis for four stages with notable environmental impact factors. Comparative analyses with coal-fired and conventional natural gas power generation was conducted based on dimensionless literature data. The following insights serve as a foundation for informed project decision-making, fostering the growth of the industry.

1. When the study case outputs 1 kWh of electricity, the operation phase had the highest environmental impact in the study's life cycle, comprising 78.37% of the total combined environmental impact, followed by the fuel production phase with 15.55%, and the fuel transport phase and the project construction phase with 3.87% and 2.21%. Specifically, the operation phase contributed the largest proportion of GWP, EP, AP, ADP, and HTP impact potentials, which were 81.82%, 71.90%, 56.03%, 82.97%, and 55.19%, respectively. The POCP impact potential was mainly tied to the fuel production phase, with the construction phase contributing the least to each impact. The possible reasons were identified as the large amount of gas released by combustion and the consumption of natural gas during the operation phase for the most serious environmental.
2. Sensitivity analyzes highlighted power generation efficiency (0.75) and natural gas consumption (0.62) in the operation phase as critical factors. Recommendations for operational projects include utilizing clean energy and advanced technology, collaborating with gas enterprises for effective gas supply, implementing energy monitoring and control for optimized operations, and enhancing the operation and maintenance of core equipment while strictly regulating economic, social, and safety aspects associated with gas facilities. For DES projects in the planning stage, recommendations include actively collaborating with local governments to create an integrated energy system based on local resources, optimizing system configuration for maximum efficiency, and prioritizing research and development in core technologies and instruments, including gas internal combustion engines and turbines.
3. Based on dimensionless data from the case study and literature using the CML2001 method, we found that GWP was the primary environmental impact in CPG systems and ADP in CNGPG and the discussed DES system when illustrating the combined environmental impacts of CPG and CNGPG for 1 kWh of electricity output. The study revealed substantial benefits of DES in minimizing overall life cycle energy consumption and reducing greenhouse gas emissions. These natural gas distributed power generation projects can strike a balance between efficiency and environmental protection within the domestic context, underscoring the need for enhanced policy and economic support. At the national level, the analysis of China's policy direction for natural gas distributed energy focuses on increasing natural gas consumption, reducing operating costs, and facilitating grid connection. However, existing deficiencies in the policy system hinder standardized development. Recommendations include enhancing policy implementation rules, harmonizing electricity subsidies, incorporating technical standards, and expanding targeted local policies to promote institutional reform and effective support in provinces, municipalities, and regions.

In future research endeavors, an exploration of integrating the life cycle cost method into the fundamental LCA framework will be undertaken to calculate the comprehensive life cycle power generation cost of DES. This integration aims to establish a meaningful correlation between environmental impacts and economic costs, with the goal of constructing an all-encompassing evaluation system that addresses both environmental considerations and financial aspects. Additionally, we envision conducting case studies on distributed energy in diverse contexts and locations to enhance the comprehensiveness and applicability of the LCA approach. This strategic approach is intended to provide a more nuanced understanding of the system's dynamics and contribute to a more robust and widely applicable framework.

## Data Availability

The data that support the findings of this study are available from the corresponding author, [Ting Ni], upon reasonable request.
